# Exposure and Predictive Factors of Postural Development from the Perspective of the Reliability of Their Measurement Tools: A Systematic Review

**DOI:** 10.3390/children13010076

**Published:** 2026-01-03

**Authors:** Tania Mirón-Pérez, Juan Luis Sánchez-González, Víctor Navarro-López, Mónica Menendez-Pardiñas, Sanz-Esteban I

**Affiliations:** 1 Doctoral Program: Health, Disability, Dependency, and Well-Being, University of Salamanca, Patio de Escuelas Menores (Street), 37008 Salamanca, Spain; 2 Department of Medicine, Faculty of Medicine, University of Salamanca, 37007 Salamanca, Spain; juanluissanchez@usal.es; 3 Institute of Biomedical Research of Salamanca (IBSAL), 37007 Salamanca, Spain; 4 Department of Physical Therapy, Occupational Therapy, Rehabilitation and Physical Medicine, Rey Juan Carlos University, 28922 Madrid, Spain; victor.navarro@urjc.es; 5 Early Intervention and Child Rehabilitation Unit, University Hospital Complex of A Coruña (CHUAC), 15006 A Coruña, Spain; 6 Physiotherapy, Medicine and Biomedical Sciences Department, University of A Coruña (UDC), Campus de Oza, s/n, 15006 A Coruña, Spain; 7 Faculty of Medicine, Health and Sports Department of Physiotherapy, Campus de Villaviciosa, Universidad Europea de Madrid, calle Tajo s/n, Villaviciosa de Odón, 28670 Madrid, Spain; ismael.sanz@universidadeuropea.es

**Keywords:** posture, postural alignment, children, assessment tools, reliability, validity

## Abstract

Postural alignment can be influenced by intrinsic and extrinsic factors; failure to control these confounding factors and the use of invalid tools increase the risk of bias and may distort the results. **Objective:** The first objective is to identify the confounding factors that may influence the evaluation of body posture in children. The second objective is to determine which methods or tools are used to analyze postural alignment and to review the evidence regarding their validity and reliability, in order to strengthen the credibility of the results obtained. **Methods**: A systematic review was performed following the PRISMA 2020 criteria. Eligible studies were searched in the Virtual Health Library, Scopus, Medline, Web of Science, PEDro, and the Cochrane Library throughout the entire month of December 2024. Observational studies written in English, Portuguese, or Spanish that analyzed body posture (as the dependent variable) in children under 12 years of age were included. Articles not available in full text or those that assessed only a single body region were excluded. The methodological quality of the studies was assessed using the Newcastle–Ottawa scale, while the ROBINS-E tool was used to assess risk of bias. The synthesis of results was presented as a narrative review. **Results:** A total of 42 observational articles were included. No meta-analysis was conducted, and the findings are synthesized through a narrative review. The ROBINS-E tool showed a generalized result of high risk of bias, while the Newcastle–Ottawa scale reported moderate quality for longitudinal and case–control studies, with worse scores for cross-sectional studies. Methodological limitations: The differences found in the designs, population, and outcome measures generate high methodological variability that limits the possibility of quantitative synthesis. Likewise, the available evidence on the reliability of the tools is insufficient, which conditions the interpretation of the reported results. **Conclusions:** The findings with the strongest scientific support suggest that anthropometric variables or those related to body composition may be associated with body alignment. By contrast, there is still controversy regarding the influence of sex and age on postural variables. Sport modality or the weight of the school backpack could also play a role in posture; however, more high-quality studies are needed to contrast the results. The quality of the evidence is limited by heterogeneity in study designs, insufficient control of confounding factors, and the use of tools with inadequate validity and reliability. **Other:** The study was registered in PROSPERO under the number CRD42024618753. This research received no external funding.

## 1. Introduction

Static postural alignment is defined as the positional relationship between the different joints and body segments, whose underlying structures must be maintained in dynamic equilibrium to allow the greatest energy efficiency [1], whereas postural attitude is defined as the set of positions that all joints of the body can adopt at a given moment, while always keeping the center of body mass within a base of support [1,2].

Within the classical literature, Peterson Kendall et al. [1] described the differences in the postural pattern in bipedal standing of children aged 18 months compared with the adult reference standard. Other authors have argued that children’s posture begins to resemble the adult reference standard at 10 years of age, when the axial curves reach maturity [3,4].

Subsequently, various researchers have continued to study how postural alignment develops throughout growth, addressing aspects such as age-related differences or differences between sexes [5,6,7,8,9,10,11,12].

Regarding deviations from the postural pattern, Penha et al. [8] and Peterson Kendall et al. [1] warn that there are extrinsic and intrinsic factors that may affect posture, whether hereditary, environmental, or related to the individual’s physical condition, secondary to socioeconomic or emotional factors. These are known as physiological deviations, such as knee valgus or flat feet, when the child begins to stand and walk [1,3].

The presence of uncontrolled confounding factors in research increases the risk of bias and may underestimate, reverse, or overestimate the outcome under study. Therefore, it is essential to know all factors that influence body posture prior to the design phase of a research project [13].

Following these hypotheses, various researchers have sought to analyze the influence of sex [5,8,10,12]; weight, height, or body mass index [9,14,15]; motor control [9]; or sports participation [14,16] on postural alignment, and have also examined the clinical characteristics present in certain pathologies in comparison with typically developing populations [17].

To identify all the factors that affect body posture, it is necessary to conduct a systematic review that helps future researchers control confounding factors during the study design phase and that also, indirectly, allows the identification of the least studied age ranges within the pediatric population.

Currently, the evaluation techniques taught to physiotherapists in training are based on qualitative visual observation to identify altered postural patterns; such techniques are not supported by evidence-based practice (EBP) [1,6]. EBP holds that clinical observations should be supported by quantitative data that correlate with them [18].

Measurement instruments may have limitations in accurately measuring a given event. Inadequate sensitivity or specificity will increase the risk of obtaining false negatives or false positives for the event of interest [13]. If a study uses a non-valid instrument to measure the association between a variable and body posture, it may generate biased results that do not reflect the true relationship between both variables.

Therefore, the veracity of the data presented will depend on the reliability of the tool used and on the methodological quality of the study.

The gold-standard tool for quantitative measurement of posture is radiography [7]. However, in 2012, SOSORT (Society on Scoliosis Orthopaedic and Rehabilitation Treatment) supported the use of non-invasive assessment devices as part of scoliosis evaluation to reduce the frequency of radiation exposure [19]. New non-invasive and less costly tools for postural assessment continually emerge [7,8], and some authors have conducted reliability analyses of them [9].

At present, there are no systematic reviews that comprehensively evaluate the factors influencing body alignment from the perspective of the scientific support of the measurement tools employed. In this context, conducting a systematic review with meta-analysis is complex, as it requires high methodological quality, adequate control of confounding factors to reduce heterogeneity, as well as the use of appropriate measurement tools. Although conducting systematic reviews with meta-analyses focused on a single factor affecting body posture may be methodologically simpler, the control of confounding factors and the reliability and validity of measurement tools continue to represent an obstacle.

All of the above leads us to ask: In an observational study on body posture, what confounding factors might arise? What types of tools have been used to conduct postural analysis in the child population under 12 years of age, where adolescence begins according to Mansilla [20]? Have these tools demonstrated validity and reliability, or could they affect the results of the outcome of interest?

- Therefore, the first objective of this work is to define which confounding factors should be taken into account during postural evaluation in children up to 12 years of age so as not to distort or bias the results. The second objective is to identify which methods or tools have been used to analyze postural alignment and to determine which have previously demonstrated reliability and validity, in order to underscore the truthfulness of the reported results—an innovative approach in the present systematic review.

## 2. Materials and Methods

This systematic review followed the PRISMA checklist [21] (Preferred Reporting Items for Systematic Reviews and Meta-Analyses) and is registered in PROSPERO under number CRD42024618753. The PRISMA checklist details are presented in Table S1 in the Supplementary Material.

### 2.1. Sources and Search Methods

The search was conducted throughout the entire month of December 2024, from the 1st to the 31st, in the following databases: Virtual Health Library (BVS), Scopus, Public MEDLINE (PubMed), Web of Science (WOS), PEDro (Physiotherapy Evidence Database), and the Cochrane Library. Boolean operators (OR and AND) were used as search strategies, and parentheses were used as a proximity operator, establishing the following search formulas for the first and second review:

- (postural alignment OR postural analysis OR postural evaluation OR postural assessment AND children)
- (posture AND children).

The search was refined within the databases. The search boxes used are provided in Appendix S2 in the Supplementary Material.

### 2.2. Study Eligibility Criteria

With the aim of detailing the factors that have been studied to analyze their possible relationship or association with postural development—considered the dependent outcome parameter—such as anthropometric measures, age, sex, among others; and of listing the available and used assessment tools (postural assessment software, psychometric scales, manual tools, etc.), the PICO strategy was applied [22]. For the formulation of the main scientific questions—and given that the objective of the study was not to find a specific exposure factor—‘P’ corresponds to the pediatric population, ‘I’ indicates that the included studies had to carry out an assessment of exposure, ‘C’ indicates that there may or may not be a comparator or control group, ‘O’ indicates the dependent variable to be measured (body posture).

#### 2.2.1. Inclusion Criteria

- No restriction by publication date in order to identify all possible analyzed factors.
- Language: English, Portuguese, or Spanish. Within search engines, the language with the highest percentage of results is English.
- Study type: descriptive or analytical observational studies.
- Full-text studies.
- Study characteristics by topic: studies that analyzed the subject’s postural pattern in the bipedal or sitting position, and those referring to postural assessment scales or tools under validation or already validated.
- Regarding sample characteristics: studies that assessed only the child population under 12 years of age.
- Without restriction by methodological quality of the studies. In order to avoid excluding studies of low methodological quality that may have used a different tool than those employed in studies with higher quality, and to prevent selection bias for our second objective, this restriction was not applied.

#### 2.2.2. Exclusion Criteria

- Study type: systematic and literature reviews; case studies or case series with a sample size ≤ 10 children per group; or clinical trials.
- Sample characteristics: children with defined pathology or symptomatology (with the exception of musculoskeletal conditions such as scoliosis).
- Articles not available in open full text.
- Study topic: observational studies that assessed postural control/balance, motor development, etc., in relation to the postural pattern when the latter was the independent variable. Also excluded were studies that assessed a single body region (e.g., neck, head, feet), except for those that assessed the trunk, given its extent and the number of joints involved, as the objective was to understand global postural attitude.

### 2.3. Data Extraction and Analysis Methodology

Title and abstract screening, as well as data extraction, were carried out by two investigators (TMP and ISE). In cases of discrepancies, a third investigator (JLSG) intervened. After a thorough full-text reading of each study to determine whether the inclusion criteria for inclusion in the review were met, studies were classified by design: descriptive and analytical observational studies, and cross-sectional studies assessing reliability or validity. Duplicate records were reviewed and removed manually by comparing the results. Subsequently, a joint table of sample characteristics and study objectives was developed to identify factors that could affect body posture. The tools used in the different observational studies included in the review were also examined and described in detail. The reference lists of the included observational studies were used to identify reliability and validity studies of the tools employed that had not appeared in the initial search process; when this information was not available, a manual search was conducted. Finally, the results obtained from the various cross-sectional reliability and validity studies identified were discussed descriptively, with the aim of providing information on potential bias due to a lack of instrument sensitivity that may have occurred in the observational studies identified within the specified period. For the descriptive analysis of the data, a table was prepared including the following information: authors and reference, study design, sample, main study variables, outcome tools, and results.

Two independent reviewers assessed the quality and risk of bias of the included studies using the same methodological process. Inter-evaluator reliability was evaluated using the kappa coefficient (>0.7 indicating high agreement, 0.5–0.7 moderate agreement, and <0.5 low agreement).

Due to the considerable heterogeneity in study designs, sample characteristics, outcome measures, the different instruments used, and the high risk of bias, no meta-analyses or additional analyses were conducted. The results were presented through a narrative synthesis, as the variability in the validity and reliability of the tools makes comparison between studies difficult and limits confidence in the results. Therefore, the evidence was not assessed using the GRADE system or similar tools. At present, the inclusion of meta-analyses in future updates is not anticipated.

### 2.4. Assessment of Methodological Quality

The quality and methodological validity of the selected studies were independently assessed by two reviewers.

The Newcastle–Ottawa Scale (NOS) was used to assess the quality of non-randomized studies and the risk of bias (case–control, prospective, and cross-sectional studies) included in this review. Scores from all categories were summed. An adaptation of the scale for cross-sectional studies was used [23]. Studies obtaining 8–9 stars were considered high quality or low risk of bias; studies scoring 5–7 stars were considered to have moderate risk; and studies scoring fewer than 5 were deemed to have a high risk of bias and therefore lower quality, according to the Academic Advisory Network on Systematic Reviews as stated in the PAPIME PE203421 Project [23].

### 2.5. Risk of Bias Assessment

The risk of bias assessment of the selected studies was conducted independently by two reviewers.

The ROBINS tool (Risk Of Bias In Non-Randomized Studies) was used to assess the risk of bias in non-randomized studies. Specifically, ROBINS-E was employed [24], which aims to evaluate the risk of bias in the outcomes of observational epidemiological studies through a series of questions covering seven domains of bias. Based on the responses, judgments are made by domain (low risk, some concerns, high risk, or very high risk), with an overall judgment of the risk of bias in the outcome.

Before using ROBINS-E in a systematic review, the reviewer must specify the important confounding factors that are likely to influence the association between exposure and outcome. With respect to body posture, these factors are the subject’s age and sex, factors related to body composition, and sports participation.

### 2.6. Extracted Variables

- General study characteristics

Author and year of publication
Study design
Sample size

- Population characteristics

Age or age range within the pediatric population
Sex
Subdivisions based on specific parameters according to the type of study (anthropometric variables, presence or absence of postural alterations, age, sex, etc.)

- Posture-related variables

Postural parameters assessed
Assessment position (standing or sitting)

- Measurement instruments

Type of instrument used
Reported validity and reliability

- Predictive and confounding factors under study (objective)

Age and sex
Anthropometry (weight, height, BMI) and body composition–related variables
Physical activity
Ergonomic factors
Environmental factors
Other factors

- Main outcomes

Relationship between postural variables and the factors studied
Secondary associations identified

## 3. Results

Results were presented descriptively in tables, with no meta-analysis or additional analyses.

The included studies exhibited substantial inter-study heterogeneity. Differences were observed in the characteristics of the populations evaluated (age ranges, sex, subdivisions based on anthropometric characteristics in some studies, environment-based classification in one study, and subdivisions according to physical activity levels, among others), in the study designs (cross-sectional, case–control, longitudinal), and in the assessment procedures employed. Likewise, the tools used for postural evaluation were highly diverse and demonstrated varying levels of validity and reliability, which hinders direct comparability across studies. Additionally, the outcome measures reported varied widely, ranging from angular measurements between body points to symmetry indices or linear distances. Due to this clinical, methodological, and measurement heterogeneity, performing a meta-analysis or quantitative synthesis was not appropriate.

### 3.1. Study Selection

The database search was carried out in December 2024 and yielded a total of 7259 records. Based on the information provided by the abstracts, 7016 articles were excluded prior to screening—either because they assessed only postural control/balance, gait, psychomotor development, ranges of motion, etc.; because their study design did not match the inclusion criteria (e.g., clinical trials or reviews); or because they were duplicates.

Subsequently, after screening, 176 articles were excluded for not meeting the inclusion criteria for sample characteristics (such as age greater than 12 years) or for assessing only a single body part, according to information provided by the abstract or, in some cases, after consulting certain sections or tables of the article itself. Four articles were excluded due to study type or language, and four were excluded because the variable of interest was evaluated as the independent variable. Fifteen articles were not available in full text for analysis.

**Figure 1:**
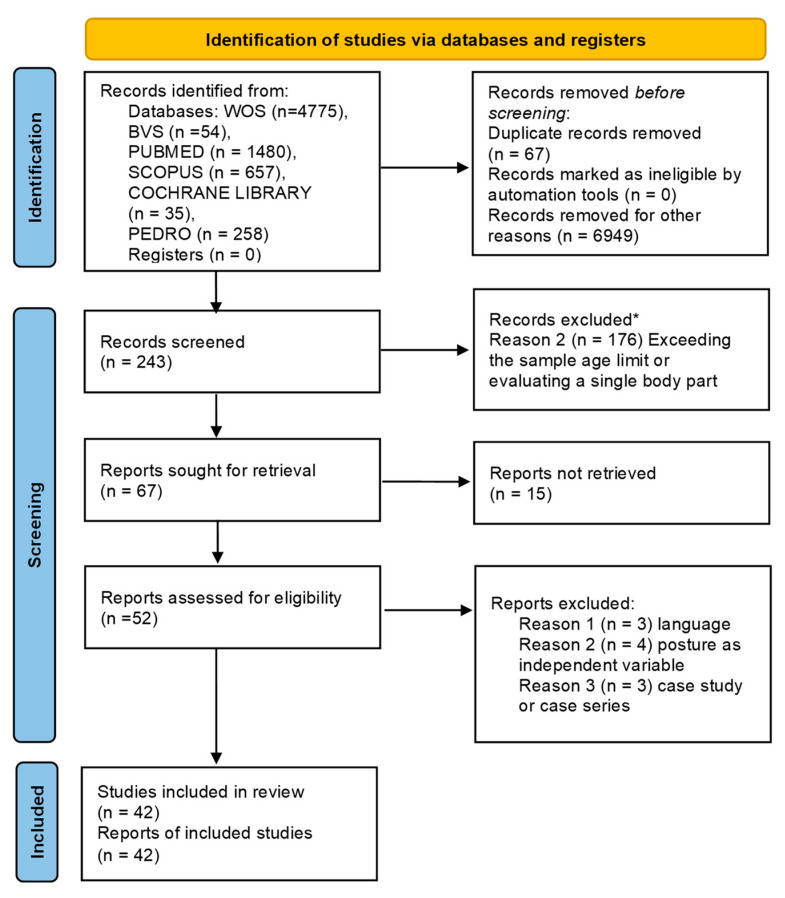
PRISMA 2020 study selection flowchart. * excluded by title and abstract.

A total of 42 articles remained for inclusion in the review [6,7,8,9,11,25,26,27,28,29,30,31,32,33,34,35,36,37,38,39,40,41,42,43,44,45,46,47,48,49,50,51,52,53,54,55,56,57,58,59,60,61]. A PRISMA flow diagram [21] was used to illustrate the selection process, which is shown in Figure 1.

### 3.2. General Characteristics of the Included Studies

The objective of a small percentage of studies was purely descriptive; among them, Santonja-Medina et al. [50] and Araújo et al. [51] aimed to describe the different postural patterns shown by the child population when adopting various positions to create standards of integral morphotypes. Other studies [6,38,45,46,54,57,58,59] focused on describing postural normative values, both quantitative and qualitative, or analyzing the prevalence/incidence of postural defects in the pediatric population without studying the interaction with other variables. The study by Jankowicz-Szymanska et al. [42] investigated not only the incidence of postural defects but also the relationship between different body structures in the presence or absence of a postural defect.

Regarding covariates, on the one hand, studies on the general population have examined the effects of external factors on the postural alignment of school-age children. The factors evaluated were demographic environment [40], physical activity or sedentary behavior [27,36,44,48,61], and the carrying of school supplies or certain ergonomic aspects [33,39,41,56,60].

On the other hand, the possible influence of intrinsic factors such as bone mineral density, body composition, body mass index, height, or body weight on postural pattern has also been investigated, either as a primary or secondary objective [9,34,42,43,47,49,51,52,54,58].

The association or relationship with the variables of age [6,7,8,9,35,37,38,45,47,53,55,57,58] and sex [8,35,37,38,42,44,45,46,49,50,51,52,54,55,58,60] has also been analyzed, both directly as a main objective and secondarily within the study.

Regarding other possible causal relationships, Walicka-Cuprys et al. [53] evaluated whether the presence of postural asymmetries in school children when adopting standing and sitting positions was related to prematurity at birth as a possible predictive factor. Similarly, Cejudo et al. [25] focused on showing how lower-limb ROM (range of motion) predicts axial misalignments, while Zmyslna et al. [26] studied the relationship between the width of the linea alba and spinal misalignments.

Regarding the characteristics of longitudinal studies, follow-up periods ranged from a maximum of 7 years to a minimum of 10–11 months, except for McEvoy et al. [9], whose two assessments were conducted within the same hour.

The main characteristics of all included studies are listed in Table S3 in the Supplementary Material.

### 3.3. Sample Characteristics of Different Studies

The various observational studies analyzed show variability in the characteristics of the selected samples according to each study’s objectives.

- Age

The age ranges of the children and preadolescents studied varied from one month after birth as the minimum age to 12 years as the maximum age. In some studies [6,7,8,9,38,45,47,53,55,57,58], as reported in the text or shown in statistical tables, children were divided into age ranges to assess the influence of age on postural patterns. The study by Zietek et al. [47] divided children according to school grade level, which represents division by age. In longitudinal cohort studies [34,35,37], children were evaluated simultaneously at different ages, allowing the study of the influence of time on posture.

- Sex

Regarding the sex of the analyzed population, all studies included children of both sexes except those by Penha et al. [6] and the case–control study by Guimarães et al. [27], which studied posture only in girls. Many cross-sectional studies divided children into gender groups [8,38,42,44,45,46,49,50,51,52,53,54,55,58,60,61], as was done for age.

- Anthropometric characteristics

In all studies, descriptive sample characteristics such as the child’s weight and height were measured (except Lafond et al. [7] and Penha et al. [57] to analyze sample homogeneity/heterogeneity. In some studies, BMI (Body Mass Index) was calculated but no grouping was done according to these characteristics, except for Brzezinski et al. [54], who used the International Obesity Task Force (IOTF) classification; Balkó et al. (44) who followed the categories described by Vignerová and Bláha (2001) [62]; Zurita Ortega et al. [58] who used tables from the en-Kid study. Notably, Jankowicz-Szymanska et al. [42], Labecka et al. [35] and Guimarães et al. [27] excluded samples based on normal BMI reference values to limit the effect of weight on posture.

- Environmental factors

Regarding intersubject subdivisions, one study [40] classified children according to whether they lived in urban or rural areas to analyze the influence of the environment on posture.

- Physical and sports activity

Others (36,48) divided children by weekly physical activity level or sport participation. In this regard, a case–control study [27] established two groups based on weekly sports practice.

- Ergonomic parameters

In the school setting, Walicka-Cuprys et al. [41] divided children according to the weight of their school backpacks.

- Other types of groupings or characteristics selected for the sample population

A longitudinal study [35] divided children according to the improvement of body posture over time to better compare changes.

Regarding other specific intrasubject characteristics, one study [51] grouped the sample based on postural models, establishing three types: swaying, flat, and neutral-hyperlordotic posture.

Heck et al. [11] classified infants according to Alberta Infant Motor Scale (AIMS) results as typically developing or motor delayed to analyze the influence on postural development. Another case–control study [53] classified children according to birth characteristics—whether full-term/preterm (>36 and <42 gestational weeks) or premature (<32 weeks)—to analyze the influence of prematurity on postural development during childhood. Meanwhile, Zmyslna et al. [26] set limits for the linea alba width to define case and control groups. The case–control study by Cejudo et al. [25] divided children by the presence/absence of thoracic and lumbar misalignment.

It is noteworthy that among cross-sectional studies analyzing the reliability of tools, Sacher et al. [28] established inclusion criteria of adjusted age from 14 to 24 weeks and the presence of postural or movement asymmetry.

### 3.4. Tools Used in the Evaluations of the Included Studies

This review found studies [6,44,45,47,56,57,58,59] that used subjective visual assessment methods, such as the postural description established by Kendall, with or without the Adams test—that is, based on conventional physiotherapeutic examination [6,47,57,58]—and others such as the Kasperczyk visual point scale [45], the Jaroš and Lomíček test [44] or methods based on Klein, Thomas, and Mayer’s methodology [59]. Other authors, as detailed in the next paragraph, used different postural assessment tools, ranging from manual to technological instruments that attempt to quantify posture. Subjective evaluation methods using scales were also found, such as the New York Posture Rating Chart [56] and the Symmetry Score for Infantile Postural and Movement Asymmetries [28].

Among the classic manual quantitative postural measurement tools using surface contact devices were the arcometer, scoliometer, inclinometer, graduated ruler, and goniometer, used in a few studies [25,30,33,36,50,54,55].

With technological advancement, digital short-distance photogrammetry began to be used in postural assessment, facilitating the improvement of surface topography tools and the development of new 2D, 3D, and 4D biomechanical analysis tools using different systems (raster stereography or ultrasound-based analysis).

Regarding digital photogrammetry, some studies used image analysis software such as CorelDraw [8,27] and ImageTool UTHSCA [9], while others used software designed for body posture image analysis, such as SAPO [31,34,46,51,52], SCODIAC [29] and BIOTONIX [7], or 3D video motion analysis systems such as APAS [11] and KINOVEA [39].

Photogrammetry enabled the sophistication of posture study tools such as Moiré surface topography [32,35,37,40,48,53,60,61] or the DIERS system [26,38,43,49], an optical light scanning method.

Other studies [41,42] used biomechanical analysis tools based on ultrasound positioning.

Regarding validation study results, the Symmetry Score for Infantile Postural and Movement Asymmetries [28] showed high internal consistency, high specificity, but moderate sensitivity (62.5%). As for manual and technological tools, only Scoliotic Diagnostic (SCODIAC) [29] showed excellent intra-observer reliability and good to excellent inter-rater reliability; however, no concurrent validity studies with radiography were found. Reported results indicate that the arcometer [30] and SAPO software [31] are also not suitable for monitoring children’s axial postural alterations. The study by Drzal-Grabiec et al. [32] using Moiré Topography (MORA System 4th Generation) reported high conformity without significant differences between the two therapists’ measurements, except for parameters evaluating torsion or asymmetry in scapular and pelvic positioning, such as shoulder line and pelvic rotation (*p* = 0.0000).

The Summary of Reliability and Validity Findings for Postural Assessment Measurement Tools is listed in Table S4 in the Supplementary Material.

### 3.5. Results on the Methodological Quality of the Included Studies

The Newcastle–Ottawa Scale (NOS) scores are detailed in Table S5, in the Supplementary Material.

Items not reported in the studies were scored as “NO.” The methodological quality of longitudinal cohort studies ranged from 7 (maximum) to 3 (minimum) out of 9 possible points, with an average quality or moderate risk score of 5.5. In case–control studies, scores ranged from 7 (max) to 3 (min) out of 9, with an average of 5.25. Finally, in cross-sectional studies, methodological quality ranged from 4 (max) to 3 (min) out of 7, with an average of 2.4. Tool validity and reliability analysis studies had an average quality of 3.

It is worth noting that in item 3 (“Exposure Determination”), none of the assessment tools used can replace the gold standard, but those tools for which concurrent validity studies were found were scored “YES,” as most tools lack such analyses. The absence of bibliographic references in the reviewed studies regarding the validity of the method used for postural evaluation interferes with NOS-based analysis. In item 6 (“Statistical Test”), a “YES” was marked only when both confidence intervals and *p*-values were present.

Regarding the quality of the observational studies analyzed, NOS results show higher quality for longitudinal and case–control studies, indicating moderate quality. Cross-sectional studies obtained lower scores overall.

The reliability analysis showed high agreement between the two reviewers regarding the methodological quality of the included studies (kappa = 0.87).

### 3.6. Results on the Risk of Bias of the Included Studies

**Figure 2:**
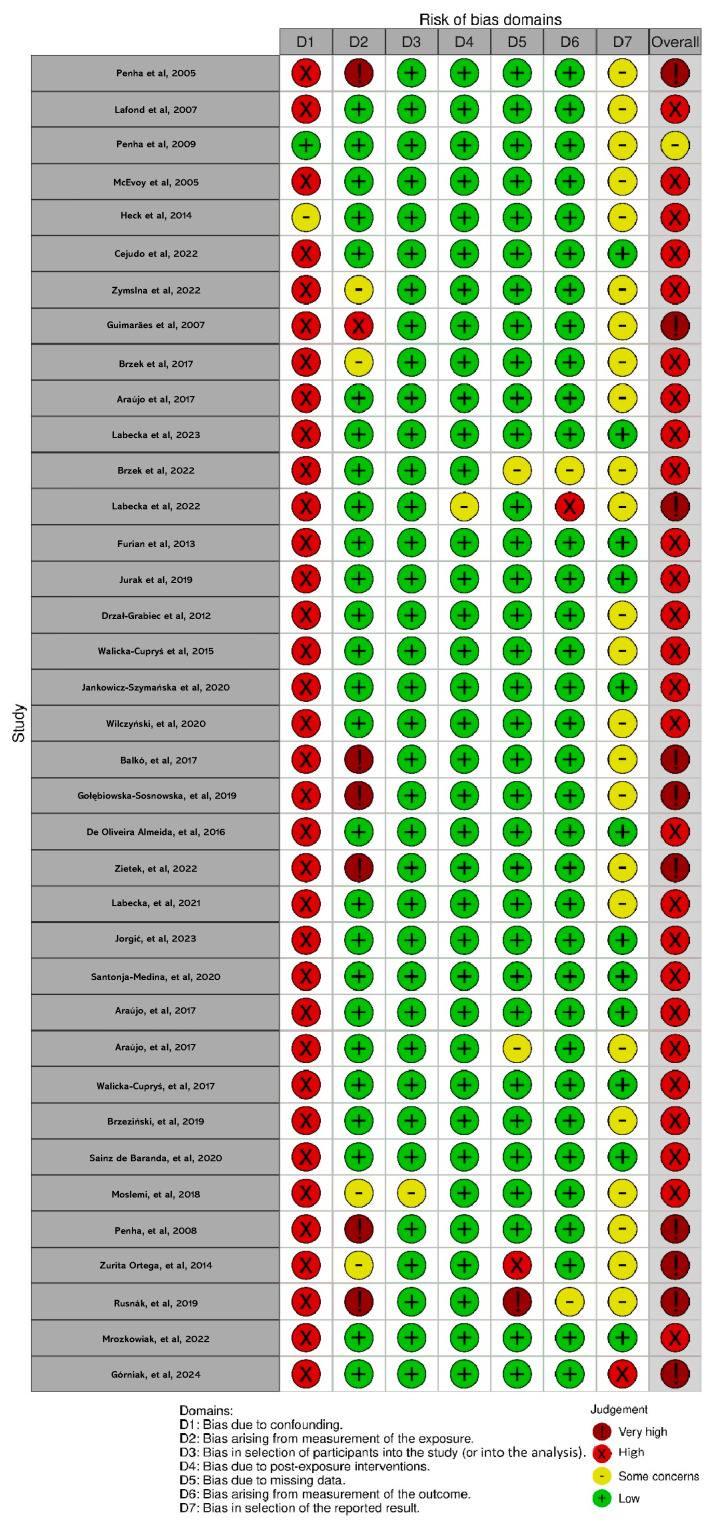
Risk of bias for observational studies with the ROBINS-E tool and visualized with the robvis tool for studies included in the systematic review [24]. Domains: (D1) Bias due to confounding; (D2) Bias arising from measurement of the exposure; (D3) Bias in selection of participants into the study (or into the analysis); (D4) Bias due to post-exposure interventions; (D5) Bias due to missing data; (D6) Bias arising from measurement of the outcome; (D7) Bias in selection of the reported result [6,7,8,9,11,25,26,27,33,34,35,36,37,38,39,40,41,42,43,44,45,46,47,48,49,50,51,52,53,54,55,56,57,58,59,60,61].

Figure 2 presents the results obtained using the ROBINS-E tool, showing that Domain 1 presented a generally high risk due to the presence of confounding factors that were not adequately controlled. Several literature reviews highlight the importance of implementing confounding bias control strategies in both observational and experimental studies, given their potential impact on research outcomes [63,64]. The validity and reliability analysis studies were excluded from the use of this tool.

Before conducting a study, it is essential to review the theoretical framework surrounding the research design; by reviewing the prior literature, potential confounding variables can be identified and controlled during project development [64].

Regarding limitations, the items addressed in the quality assessment of the articles using the Newcastle–Ottawa (NOS) scale and the ROBINS-E tool depend on the reviewers’ judgment and experience. The literature recommends their use by two reviewers reaching a consensus; however, in this case, only one reviewer performed the evaluation.

The reliability analysis showed a high level of agreement between the two reviewers regarding the risk of bias of the included studies (kappa = 0.87).

### 3.7. Results of Individual Studies in Relation to the Tools Used

Of the 42 studies included in this study, only 9 investigations used tools that are currently considered valid and reliable.

The studies by Sainz de Baranda et al. [55], Wilczynski et al. [43], Furian et al. [38], Zmyslna et al. [26], Brzek et al. [33], Brzek et al. [36], Santonja-Medina et al. [50], Cejudo et al. [25] and Jorgic et al. [49] used an inclinometer or the DIERS Formetric 4D system for postural assessment, which allows us to consider that, for those parameters that have demonstrated validity and reliability, the results were not substantially influenced by the measurement tool and that the variables of interest were appropriately assessed.

### 3.8. Results of Individual Studies in Relation to Confounding Factors

If we now focus on the topic of postural development, a trend toward quantitative studies of child posture at older ages has been observed, while such studies are scarce in children under 6 years of age and insufficient in the first year of life, when the greatest postural and motor changes occur.

Regarding the results found across the different studies, several factors were identified as being examined in relation to body alignment. Among intrinsic factors, age, sex, body composition, and anthropometry have been investigated for their association with specific postural variables. Likewise, extrinsic factors such as physical activity, demographic context, the carriage of school materials, and certain ergonomic aspects have also been studied. In addition, the probability of relationships between different body segments was reported. However, 33 studies did not use valid and reliable measurement tools, which may have influenced their findings.

If we focus on the nine studies that used appropriate measurement tools, we can summarize:

- Age as an intra-subject factor

The studies by Sainz de Baranda et al. [55] and Furian et al. [38] analyzed the influence of age on body posture. Both studies divided their samples by age and sex, and the age range evaluated in both was 6 to 11 years. Furian et al. [38] found no significant differences in spinal variables among age groups. However, Sainz de Baranda et al. [55] observed a possible relationship between age and postural variables in the sitting position.

- Sex as an intra-subject factor

The influence of sex was analyzed in the studies by Furian et al. [38], Sainz de Baranda et al. [55], and Santonja-Medina et al. [50]; the latter further classified children according to postural patterns and sex. Furian et al. [38] did not find significant postural differences between boys and girls, whereas Sainz de Baranda et al. [55] reported gender-related differences in postural alignment. Santonja-Medina et al. [50] found differences in the frequency of each postural morphotype between sexes.

- Anthropometry or body composition–related variables, as an intra-subject factor

The association between posture and anthropometry was examined by Wilczynski et al. [43], Sainz de Baranda et al. [55], and Jorgic et al. [49].

In the first two studies, children were stratified by age and sex, whereas in the latter study, the sample was subdivided only by sex. Although the analysis of the association between posture and anthropometry was not the primary objective of the study by Sainz de Baranda et al. [55], relationships between variables were observed as secondary findings. All authors found associations between anthropometric parameters and body posture.

- Relationship between body structures

With regard to other intra-subject causal factors, Zmyslna et al. [26] focused on examining whether the width of the linea alba is associated with body posture. Meanwhile, the case–control study by Cejudo et al. [25] explored the association between lower limb ranges of motion and the presence or absence of spinal misalignment according to sex. The authors found associations between the structures.

- Ergonomic variables as an extra-subject factor

The extra-subject factor of “school backpack weight” was investigated by Brzek et al. [33] in a longitudinal study that stratified children by sex. The authors found an association between posture and the extra-subject factor.

- Physical and sports activity as a factor

Regarding physical and sports activity as an extra-subject factor, the study by Brzek et al. [36] took sex into account as a confounding factor. The authors found positive effects of sports practice on body posture, regardless of sex.

## 4. Discussion

This systematic review provides an overview of the factors that affect or predict body posture alignment, the methods or tools used to assess posture, and which of these have been shown to be valid and reliable.

Regarding the results reported in the different studies, numerous possible causes influencing body alignment were found. Among the intrinsic factors, age, gender, body composition and anthropometry were identified as influencing certain postural variables. Likewise, other extrinsic factors such as physical activity, demographic situation, carriage of school materials, or certain ergonomic aspects could affect body posture. Compared with a previous systematic review conducted in the same target population [65], more factors have been identified than those reported therein, where the authors only discussed the effect of school backpack use, body composition and physical activity on body posture.

### 4.1. Reliability of the Tools

The validity and reliability of measurement tools are important to avoid biases derived from instruments, which reduce research quality [13].

To justify the criteria or minimum requirements established for the postural assessment tools analysed below, the results obtained in certain reliability studies of the gold standard tool—radiography—were consulted [66,67,68].

Four of the studies [6,47,57,58] analysing posture employed subjective visual assessment methods such as the postural description established by Kendall, the Jaroš and Lomíček test [44], Kasperczyk’s visual point scale [45] or those based on the methodology of Klein, Thomas and Mayer [59], which could call into question the results reported by the authors and their conclusions.

A study [56] used the New York Posture Rating Chart to assess postural alterations. Previous research [69] reported Cronbach’s coefficients with acceptable ratings for photographic assessments (0.77) and excellent ratings for physical examinations (0.91). However, no concurrent validity studies nor studies developed in paediatric populations were found.

With respect to the reliability of manual tools, Azadinia et al. [70] analysed the concurrent validity and reliability of the J Tech Dualer IQ digital inclinometer and the flexible ruler for measuring thoracic kyphosis. In this study, the inclinometer demonstrated excellent inter- and intra-rater reliability (intraclass correlation coefficient, ICC > 0.90) and good results (ICC > 0.80) when compared with radiography in both groups. Regarding the analogue inclinometer, another study [71] reported excellent reliability (ICC > 0.75) and a high correlation with radiographic measurements for the sagittal parameter of thoracic kyphosis in the early (r = 0.84) and late (r = 0.75) growth phases, with no significant differences observed between inclinometer and radiographic measurements (*p* > 0.05). However, for lumbar lordosis, reliability was moderate in both growth phases, with significant inter-examiner differences (*p* < 0.05), particularly in the late growth phase. In addition, concurrent validity was weak, with low to moderate correlations in the early (r = 0.38) and late growth phases (r = 0.49), and significant differences between inclinometer and radiographic measurements in both phases (*p* < 0.001).

Regarding the graduated ruler or goniometer, a previous study [72] that analysed concurrent validity for measuring the lower limb axis reported only moderate correlations (r = 0.67), which questions its use for measurement purposes. Other tools, such as the caliper used to measure intercondylar distance, obtained better scores (r = 0.89). More studies are needed on the reliability of the goniometer.

With reference to the scoliometer, the study by Coelho et al. [73] found a good correlation (r = 0.7; *p* < 0.05) with the Cobb angle based on the greatest angle of trunk rotation (ATR) measured at each vertebral level and high sensitivity of the instrument for detecting trunk rotation of 5° ATR at 87%. The results showed excellent intrarater reliability and very good interrater reliability. The authors recommend conducting further studies to measure test–retest reliability before incorporating the tool into clinical practice.

The study by Tabard-Fougère et al. [74] demonstrated good concurrent validity of the DIERS Formetric-4D system (based on body line projection) using DICAM software (r = 0.70; *p* < 0.01) for measuring the scoliosis angle, with no significant differences between methods (t = 0.53; *p* = 0.60) and a mean difference of 5.4° ± 4.2°, although in 15 of the 35 subjects the results differed by more than 5° between both methods. Intra-rater reliability was acceptable (ICC > 0.70), while inter-rater reliability was excellent, exceeding the established ICC threshold (ICC > 0.80) for all variables except the pelvic obliquity angle, which obtained low scores.

As for Moiré topography (using the MORA System 4th Generation software, concurrent validity was tested against the gravitational inclinometer [75], but not against radiography.

Concerning the concurrent validity of the Zebris CMS-HS motion analysis system (ultrasound-based) with WinSpine software, the results of one study [76] showed significant correlations with the Cobb angle for the thoracic kyphosis angle (*p* = 0.000) and excellent correlations (r = 0.95), as well as for the thoracic scoliosis angles (r = 0.85) with differences within the limit of agreement. A good correlation was found for the lumbar lordosis angle (r = 0.76), although differences outside the agreement limit appeared in patients with lumbar lordosis ≥50°, while for thoracolumbar/lumbar inclination, angles were underestimated if above 15°, though the correlation remained significant (*p* = 0.000) and excellent (r = 0.84). Nevertheless, it is important to note that the sample size consisted of 19 participants aged between 8 and 16 years. No inter- or intra-rater reliability studies were found for this tool.

Regarding short-distance photogrammetry software, specifically using Corel-Draw 11.0, previous research [77] found high internal consistency for lateral curvature angles of the thoracic and thoracolumbar segments (Cronbach’s α > 0.90). However, consistency decreased for the lumbar segment (Cronbach’s α = 0.705 for method 1 and 0.604 for method 2). The data indicate good intra-rater reliability and acceptable inter-rater reliability. No linear relationships were found between photogrammetric and radiographic measurements, affecting their validity for assessing the magnitude of lateral spinal deviation. With respect to the Corel Draw tool, its use as a complementary assessment method should be ruled out when the aim is to evaluate severe curves.

A study [78] attempted to demonstrate the concurrent validity of Kinovea software with radiography, but results did not show significant correlations in measuring the lumbar lordosis angle (*p* > 0.05) based on Pearson’s correlation coefficients. However, regarding thoracic kyphosis, validity depended on evaluator experience, with only the evaluator having over 20 years’ experience (*p* = 0.049) and an expert with five years’ experience (*p* = 0.013) providing acceptable validity. On the other hand, intra- and inter-rater validity revealed significant correlations for all angles according to ICC (*p* < 0.001). Nevertheless, it is important to note that the sample size consisted of 18 participants aged between 9 and 73 years.

Regarding SAPO software, a 2012 study [79] reported inter-rater reliability with low correlation (r = 0.13 to 0.59) in 15 variables, moderate correlation (r = 0.61 to 0.74) in 10 variables, and high correlation in 4 variables (r > 0.80), results that are inconsistent with those reported by Santos et al. [31]. No concurrent validity studies were found.

Although many instruments have been evaluated in terms of validity and reliability, several tools present important limitations, such as the absence of studies in pediatric populations, as is the case with the New York Posture Rating (NYPR) and the validity study of the MORA 4G system, or the lack of validity studies comparing the tool with a gold standard, as observed for the MORA 4G and SCODIAC systems. For other tools, such as the Biotonix, ImageTool, and APAS software, no studies evaluating validity or reliability were found, which prevents determining whether the tool measures what it is intended to measure and whether its measurements are consistent. Likewise, tools such as SAPO software, Corel Draw, and the arcometer showed limited evidence supporting their validity and reliability for pediatric postural assessment in the analyzed studies, or, in the case of Kinovea, Zebris CMS-HS, the goniometer, and the scoliometer, the available research was insufficient or larger sample sizes are required.

In contrast, the DIERS Formetric 4D system and the digital inclinometer demonstrated good levels of reliability and validity in pediatric populations. Likewise, the gravitational inclinometer showed adequate results only for the assessment of thoracic kyphosis, demonstrating good levels of reliability and validity for this parameter. Nevertheless, it is essential to continue evaluating the available tools to confirm the consistency and accuracy of the measurements, as well as to conduct validity analyses specifically in pediatric populations in order to identify potential limitations in their application.

The absence of bibliographic references regarding the reliability and validity of the method used—evaluated using a tool considered the gold standard—hindered the assessment of the methodological quality of the studies using the Newcastle–Ottawa Scale. This limitation also complicated the discussion process and the synthesis of results based on the available evidence. Therefore, conducting a thorough preliminary search during the study design phase, focused on the validity and reliability of the intended method, is essential to ensure reliable results and to improve the methodological quality of the research.

### 4.2. Evidence of Results Considering the Quality of the Tools

Considering that assessment tools such as the Diers system and the inclinometer have demonstrated good to excellent reproducibility and adequate concurrent validity, it is worth highlighting the conclusions reached in these nine studies.

In the second half of childhood, one study [38] concluded that no significant differences were found between age groups (6 to 11 years) in the study of spinal variables.

The same intrasubject variable, analysed in the sitting position, according to other authors [55], revealed a possible relationship between altered postural patterns, such as lumbar and thoracic angles, with age, with increased angles at younger ages.

Concerning the association between posture and anthropometric variables, when exploring the sitting position, Sainz de Baranda et al. [55] found that children with normal lumbar kyphosis or normal pelvic inclination were taller and heavier than those with mild or moderate curves. Similar results were found by Wilczynski et al. [43], who reported significant relationships between sagittal plane axial curve angulation and body composition (*p* < 0.05). They found significant associations between posture and factors such as fat percentage, muscle mass, body water, metabolic rate, and bone mass.

Another study [49] using the same tool found similar results, identifying significant relationships between body posture and body composition—specifically, negative inverse relationships between postural pattern and both muscle percentage and fat-free mass.

Regarding gender as an intrasubject predictor factor, there appears to be controversy among studies. Furian et al. [38] found no significant differences related to sex. Conversely, Sainz de Baranda et al. [55], using an inclinometer, found significant gender differences.

The case–control study by Zmyslna et al. [26] found a statistically significant correlation in trunk inclination, which was negative in the group with linea alba width >10 mm, indicating, according to the authors, a posterior displacement of the spinal axis over the vertical. Meanwhile, another case–control study [25] found that restricted iliopsoas and hamstring values were significantly correlated with sagittal spinal misalignments in boys for both thoracic and lumbar curves.

Regarding the extrasubject factor “school backpack weight”, Brzek et al. [33] observed, after one year of evaluation, an influence of backpack weight on postural changes, particularly in thoracic and thoracolumbar rotation variables and in plumb-line deviation, with gender differences.

On the other hand, regarding physical and sports activity, the results of Brzek et al. [36] showed that karate had positive effects on stabilising body posture, whereas other weekly physical activities were associated with greater alterations, regardless of gender.

Not only the reliability and validity of the tools influence the level of evidence; heterogeneity in study designs also affects the quality of reporting, with longitudinal and case–control studies generally obtaining higher scores in methodological quality assessments. In the previously presented studies, a high degree of heterogeneity was observed in the sample populations.

The controversy regarding the influence of gender on postural alignment could be attributed to the high risk of bias present in studies that have not controlled for all potential confounding factors. Specifically, the lack of control over variables such as body composition or level of physical activity could explain the contradictory findings. A similar situation was observed with respect to age; however, this variable may also depend on the overall body position chosen for the analysis, whether standing or seated. Nevertheless, controlling for confounding factors remains necessary to reach definitive conclusions and to determine at which stages of development posture may be influenced by age. Other variables, such as backpack weight, level of physical activity, or sport modality, could influence postural development. However, insufficient studies were identified that analyzed these factors using moderately valid tools that would allow the available results to be contrasted.

Likewise, some studies suggest a possible predictive relationship between the width of the linea alba or the ranges of motion of the hip and ankle and spinal misalignment. However, once again, studies with greater scientific support are required to confirm and contrast these findings.

### 4.3. Strengths and Limitations

The main strength of this review lies in employing two different search methodologies formed by distinct keywords related to body posture, which may have increased the range of factors identified.

As a limitation, restricting the search to the paediatric population may have excluded studies that included samples representing transitional stages between childhood and adolescence.

On the other hand, the analysis of the instruments used across the different studies allowed the identification of those with greater support from scientific evidence, constituting an additional strength.

Finally, the absence of a meta-analysis represents an important limitation due to the lack of quantitative data to support the reported results.

### 4.4. Clinical Implications of the Results Obtained

The findings suggest that anthropometric variables such as weight, height, and body composition significantly influence postural development in children. This evidence highlights the need to incorporate these variables into assessments performed by physiotherapists, particularly in school-aged children, considering how they relate to posture in order to adapt recommendations for physiotherapeutic treatment.

Furthermore, it is advisable to educate families on the benefits of physical activity and the need for appropriate use of school backpacks to prevent long-term musculoskeletal disorders.

## 5. Conclusions

This systematic review aimed to synthesize the available evidence on the factors affecting postural alignment in pediatric populations, taking into account the demonstrated validity and reliability of the tools used. Currently, radiography remains the most accurate method for diagnosing postural alterations; however, some non-invasive methods, such as the DIERS system and the digital inclinometer, have demonstrated good to excellent reproducibility, with satisfactory results compared with radiography. Regarding the analogue inclinometer, it showed adequate results only for the assessment of thoracic kyphosis, demonstrating acceptable reliability and validity for this parameter.

Regarding the findings of studies with greater scientific support, derived from the use of moderately valid tools, anthropometric variables, or those related to body composition may be associated with body alignment. Controversy persists regarding the influence of gender on postural variables. A similar situation is observed with respect to age; however, this variable also appears to be conditioned by the overall body position adopted for the analysis, distinguishing between standing and seated positions. Nevertheless, further studies controlling for confounding factors are required to reach definitive conclusions.

Other variables, such as backpack weight, physical activity, or sport modality, could influence postural development, although the available evidence is limited.

The quality of the evidence is limited by heterogeneity in study designs, insufficient control of confounding factors, and the use of tools with inadequate validity and reliability. We also identified that postural development has been mainly studied in older children, with scarce research in children under 6 years of age.

Future studies should prioritize longitudinal designs using appropriate tools and controlling for all confounding factors in children, including those under six years of age. In addition, validation studies of assessment tools in pediatric populations or the development of new tools are also needed.

## Data Availability

To the corresponding author by email.

## Supplementary Materials

The following supporting information can be downloaded at: https://www.mdpi.com/article/10.3390/children13010076/s1, Table S1: PRISMA checklist details, Appendix S2: Search Strategies Used, Table S3: Main characteristics of the studies, Table S4: Summary of Reliability and Validity Findings for Postural Assessment Measurement Tools, Table S5: Details of the Newcastle-Ottawa scale score. Analysis of observational case–control and cohort studies, adapted to cross-sectional studies.

## Abbreviations

The following abbreviations are used in this manuscript:

EBP	Evidence-based clinical practice
SOSORT	Society on Scoliosis Orthopaedic and Rehabilitación Treatment
PRISMA	Preferred reporting items for Systematics Reviews and meta-analyses
BVS	Virtual Health Library
WOS	Web of science
NOS	The Newcastle-Ottawa Scale
ROBINS	Risk Of Bias In Non-Randomized Studies
ROM	Range Of Motion
BMI	Body mass index
IOTF	International Obesity Task Force
AIMS	Alberta Infant Motor Scale
máx	maximum
mín	minimum
ICC	Intraclass Correlation Coefficient
ATR	Angle of trunk rotation
