# Supplementary material for: Exposure and Predictive Factors of Postural Development from the Perspective of the Reliability of Their Measurement Tools: A Systematic Review
Source: Children (Basel). 2026 Jan 3;13(1):76. doi: 10.3390/children13010076 (PMC12840426; doi:10.3390/children13010076)
Supplement: Supplementary file 1 [file children-13-00076-s001.zip › supplementary material S2.pdf]

Appendix S2: Search Strategies Used

Boolean operators (OR and AND) were used as search strategies in the databases, and parentheses were employed as proximity operators, establishing the following search formulas for the first and second reviews:

- (postural alignment OR postural analysis OR postural evaluation OR postural as-sessment AND children)
- (posture AND children).

The search was refined within the databases. The search strategies used in each database are described below:

- Virtual Health Library: Limited by main subject "posture", study type "prevalence, systematic reviews, incidence, evaluation, observational", sample age "child, preschool, infant", and document type "article".
- PubMed (Public MEDLINE): Limited by age ("newborn, infant 0–23 months, child, preschool"), languages ("English, Portuguese, Spanish"), and full-text availability.
- Scopus: Limited by subject area ("health professions"), document type ("article, review"), keywords ("child, posture, disability evaluation"), and languages ("English, Portuguese, Spanish").
- Web of Science: Limited by research area ("pediatrics, rehabilitation, orthopedics"), document type ("article"), and languages ("English, Portuguese, Spanish").
- Cochrane Library: Limited to Cochrane Reviews.
- PEDro (Physiotherapy Evidence Database): Used keywords "postural alignment", "postural evaluation", "postural analysis", "postural assessment", and "posture", truncated with (*) and limited by "Systematic review/Clinical trial" and subdiscipline "pediatrics".
