# Supplementary material for: Exposure and Predictive Factors of Postural Development from the Perspective of the Reliability of Their Measurement Tools: A Systematic Review
Source: Children (Basel). 2026 Jan 3;13(1):76. doi: 10.3390/children13010076 (PMC12840426; doi:10.3390/children13010076)
Supplement: Supplementary file 1 [file children-13-00076-s001.zip › supplementary material S3.pdf]

TABLE S3: *Main characteristics of the studies*

Study	Design	Sample	Main study variables	Outcome tools	Result/conclusion
Cejudo et al. (25)	Observational case-control study	Children aged 10–12 years, grouped according to presence/absence of spinal misalignment and sex.	Lower-limb range of motion and its relationship with sagittal spinal misalignment.	Isomed unilevel inclinometer.	An association was observed between restricted iliopsoas and hamstring movement and sagittal spinal misalignment in boys.
Zymslna et al. (26)	Observational case-control study	Children aged 6–9 years. Groups based on the width of the linea alba.	Relationship between linea alba width and axial spinal alignment.	Diers Formetric 4D system; SAOTE-My Lab Classic-type ultrasound.	A correlation was observed between linea alba width and the position and depth of the lordotic apex as well as trunk inclination.
Brzęk et al. (33)	Prospective cohort study	Children aged 7-9 years, stratified by sex, followed for 10–11 months.	Association between school backpack weight and its characteristics with body posture.	Pedi-scoliometer; digital inclinometer; plumb line; measuring tape; scale.	A significant increase in postural alterations was observed over the course of one year. Increased backpack weight caused changes in body posture (especially trunk rotation). Strap asymmetry also affected posture.
Brzęk et al. (36)	Prospective observational study	Children aged 9–12 years followed for 11 months; divided into karate vs. other activities; stratified by sex.	Association between posture and karate practice.	Pedi-scoliometer; digital inclinometer; plumb line.	They observed a better postural evolution in children who practiced karate. A decrease in trunk rotation was observed in children who practiced karate.
Furian et al. (38)	Cross-sectional study	Children aged 6–11 years, stratified by age and sex.	Changes in pelvic position with age and sex.	Diers Formetric 4D system.	No significant differences in pelvic position by age or sex.
Wilczyński et al. (43)	Cross-sectional study	Children aged 10–11 years, stratified by age and sex.	Relation between axial spine posture and body composition.	Diers Formetric 4D system; bioelectrical impedance analysis with TANITA MC-780.	Significant associations found between posture and body composition (fat mass, muscle mass, etc.). Children with greater muscle mass more frequently exhibited normal sagittal spinal curvatures, whereas leaner children showed a higher

					prevalence of postural alterations.
Jorgić et al. (49)	Cross-sectional study	Children aged 11 years, stratified by sex.	Influence of body composition parameters on postural disorders.	Diers Formetric 4D system; body composition analyser InBody 770 instrument.	Higher muscle mass and fat-free mass decreased the risk of alignment deviations.
Santonja-Medina et al. (50)	Cross-sectional study	Children aged 8–12 years. The sample was subdivided according to three body positions. They compared the frequency of each morphotype between sexes.	To describe body posture and define integral sagittal morphotype by sex.	Isomed unilevel inclinometer. Electronic scale (OMRON BF 500)	Results support including seated assessment and forward trunk inclination for a correct evaluation protocol of the sagittal morphotype. No significant sex differences were found for the thoracic morphotype, whereas significant differences were observed for the lumbar morphotype.
Sainz de Baranda et al. (55)	Cross-sectional study	Children aged 8–12 years, stratified by age, sex, sagittal alignment categories in the sitting position, and the presence or absence of back pain.	To analyse sagittal spinal morphotype by sex, age, and incidence of back pain.	Isomed unilevel inclinometer; Goniometer for the lumbosacral angle (LSA); 8-item back-pain questionnaire; Electronic scale (OMRON BF 500)	Significant spinal (thoracic and lumbar) and pelvic differences found by sex and age. Boys presented significantly greater thoracic angles and a higher degree of lumbar kyphosis compared to girls. Likewise, greater lumbar and thoracic kyphosis angles were observed at younger ages, although the latter parameter showed a non-linear pattern with age. No association was found between posture and back pain. Children with normal lumbar kyphosis or pelvic inclination were found to be taller and heavier than those with mild or moderate curves.

Fourth dimension analysis (4D); lumbosacral angle (LSA).
