# Supplementary material for: Exposure and Predictive Factors of Postural Development from the Perspective of the Reliability of Their Measurement Tools: A Systematic Review
Source: Children (Basel). 2026 Jan 3;13(1):76. doi: 10.3390/children13010076 (PMC12840426; doi:10.3390/children13010076)
Supplement: Supplementary file 1 [file children-13-00076-s001.zip › supplementary material S4.pdf]

TABLE S4: *Summary of Reliability and Validity Findings for Postural Assessment Measurement Tools*

Study	Tool / Instrument	Type of Measurement	Population Evaluated	Reliability Type	Reliability Results	Validity Type	Validity Results
Azadinia et al. (70)	J Tech Dualer IQ Digital Inclinometer and Flexicurve	Angle of thoracic kyphosis. The adolescents were evaluated in a standing position.	A total of 105 subjects were evaluated: 81 adolescents/ young adults (aged 10 to 30 years), 3 adults (aged 30 to 50 years) and 21 older adults (aged 50 to 80 years).	Reliability (intra- and inter-rater) was assessed using ICC.	Digital inclinometer: high intra- and inter-rater reliability (ICC > 0.90) in both age groups.<br><br>Flexicurve: ICC = 0.68 (interrater, 10–30 years), 0.85 (interrater, 50–80 years), 0.86–0.87 (intrarater).	Each instrument was compared with the radiographic Cobb angle using ICC. Agreement between measurement methods was assessed using Bland–Altman analysis (identity plot + difference plot)	Digital inclinometer: acceptable concurrent validity with Cobb angle (ICC > 0.80) in both age groups.<br><br>Flexicurve: poor validity for thoracic kyphosis in both age groups. (ICC < 0.60).
Schmidt et al. (71)	Manual inclinometer (Tozz magnetic base).	Angle of thoracic kyphosis and lumbar lordosis. The adolescents were evaluated in a standing position.	39 adolescents diagnosed with idiopathic scoliosis with a Cobb angle between 20 and 40° (aged 10 to 18 years). The adolescents were divided into two groups according to the Risser sign.	To assess the intra- and inter-examiner reliability, the intraclass correlation coefficient (ICC) was used.	Excellent intra- and inter-examiner reliability was observed for thoracic kyphosis across both growth phases (ICC > 0.75) whereas lumbar lordosis showed moderate reliability (ICC = 0.47–0.66) with significant inter-examiner	To analyze concurrent validity with radiography, Spearman's correlation coefficient was used, while the Wilcoxon test was employed to analyze differences between instruments.	The concurrent validity of the inclinometer was high for thoracic kyphosis in the early (r = 0.84) and late growth phases (r = 0.75). In contrast, concurrent validity for lumbar

					differences (p < 0.05).		lordosis was low to moderate (r = 0.38–0.49), with significant differences compared with radiographic measurements (p < 0.001).
Tabard-Fougere et al. (74)	DIERS Formetric 4D	Spinal parameters derived from surface anatomical landmarks in the frontal and sagittal planes.	35 patients with idiopathic scoliosis (aged 10 to 18 years).	Intraclass correlation coefficients (ICCs) were used to assess both intra-rater and inter-rater agreement, along with standard error of measurement (SEM) and smallest detectable change (SDC) calculations.	Acceptable to excellent reliability was observed, with intra-rater ICCs ranging from 0.70 to 0.97 and inter-rater ICCs from 0.84 to 0.97, except for pelvic obliquity, which showed lower reliability (ICC = 0.27–0.50).	Concurrent validity for the scoliotic angle was assessed by comparison with the radiographic Cobb angle. The study used paired Student's t-tests, Pearson correlation coefficients, and Bland–Altman analysis.	Good concurrent validity was found (r = 0.70, p < 0.01; mean difference = 5.4° ± 4.5°).

(intra) intrarater reliability; (inter) interrater reliability; (ICC) intraclass correlation coefficients; (SEM) Standard Error of Measurement; (SDC) Smallest Detectable Change Calculations.
