# Supplementary material for: Exposure and Predictive Factors of Postural Development from the Perspective of the Reliability of Their Measurement Tools: A Systematic Review
Source: Children (Basel). 2026 Jan 3;13(1):76. doi: 10.3390/children13010076 (PMC12840426; doi:10.3390/children13010076)
Supplement: Supplementary file 1 [file children-13-00076-s001.zip › supplementary material S5.pdf]

Table S5. Details of the Newcastle-Ottawa scale score. Analysis of observational case-control and cohort studies, adapted to cross-sectional studies.

	Selection				Comparability	Outcome/exposure			
**Study**	**1**	**2**	**3**	**4**	**5**	**6**	**7**	**8**	**Total**
Penha et al.(6)	YES	NO	NO	N/A	YES	NO	NO	N/A	2
Lafond et al.(7)	NO	NO	NO	N/A	NO	YES	NO	N/A	1
Penha et al.(8)	YES	NO	NO	N/A	YES	YES	NO	N/A	3
McEvoy et al.(9)	NO	NO	NO	NO	YES	YES	YES	NO	3
Heck et al.(11)	YES	NO	NO	N/A	YES	YES	NO	N/A	3
Cejudo et al.(25)	NO	NO	NO	NO	YES	YES	YES	NO	3
Zymslna et al.(26)	YES	YES	YES	YES	YES	YES	YES	NO	7
Guimarães et al.(27)	NO	YES	NO	YES	YES	YES	YES	NO	5
Sacher et al.(28)	NO	NO	NO	N/A	NO	YES	YES	N/A	2
Stolinski et al.(29)	NO	NO	NO	N/A	NO	YES	YES	N/A	2
Sedrez et al.(30)	NO	YES	YES	N/A	NO	YES	YES	N/A	4
Santos et al.(31)	NO	NO	NO	N/A	NO	YES	YES	N/A	2
Drzal-Grabiec et al.(32)	YES	NO	NO	N/A	NO	YES	YES	N/A	3
Brzek et al.(33)	YES	NO	NO	NO	YES	YES	YES	YES	5
Araújo et al.(34)	YES	NO	YES	NO	YES	YES	YES	NO	5
Łabęcka et al.(35)	YES	NO	YES	YES	YES	YES	YES	YES	7
Brzek et al.(36)	YES	YES	YES	YES	YES	YES	YES	NO	7
Łabęcka et al.(37)	YES	YES	YES	YES	NO	YES	YES	NO	6
Furian et al. (38)	YES	NO	YES	N/A	YES	YES	NO	N/A	4
Jurak et al.(39)	YES	NO	NO	N/A	YES	YES	NO	N/A	3
Drzał-Grabiec et al.(40)	YES	NO	NO	N/A	NO	YES	NO	N/A	2
Walicka-Cupryś et al.(41)	YES	NO	NO	N/A	NO	YES	NO	N/A	2
Jankowicz-Szymańska et al.(42)	YES	NO	NO	N/A	YES	NO	NO	N/A	2
Wilczyński et al.(43)	YES	NO	YES	N/A	YES<br>YES	YES	NO	N/A	5
Balko et al.(44)	NO	NO	NO	N/A	YES<br>YES	NO	NO	N/A	2
Gołębiowska-Sosnowska et al.(45)	NO	NO	NO	N/A	YES<br>YES	NO	NO	N/A	2
Almeida et al.(46)	YES	NO	NO	N/A	NO	YES	NO	N/A	2
Zietek et al.(47)	YES	NO	NO	N/A	YES	NO	NO	N/A	2

Łabęcka et al. (48)	YES	NO	NO	N/A	NO	YES	NO	N/A	2
Jorgić et al.(49)	YES	NO	YES	N/A	NO	YES	NO	N/A	3
Santonja-Medina et al.(50)	YES	NO	NO	N/A	NO	YES	NO	N/A	2
Araújo et al.(51)	YES	NO	NO	N/A	YES	YES	NO	N/A	3
Araújo et al.(52)	YES	NO	NO	N/A	YES YES	YES	NO	N/A	4
Walicka-Cupryś et al.(53)	NO	YES	NO	YES	YES	YES	YES	YES	6
Brzeziński et al.(54)	NO	NO	NO	N/A	YES	YES	YES	N/A	3
Sainz de Baranda et al.(55)	YES	NO	NO	N/A	YES YES	NO	YES	N/A	4
Moslemi et al.(56)	YES	NO	NO	N/A	YES	YES	NO	N/A	3
Penha et al.(57)	YES	NO	NO	N/A	YES	NO	NO	N/A	2
Zurita Ortega et al.(58)	NO.	NO	NO	N/A	YES	NO	NO	N/A	1
Rusnák et al.(59)	YES	NO	NO	N/A	NO	NO	NO	N/A	1
Mrozko- wiak et al.(60)	NO	NO	NO	N/A	YES	YES	NO	N/A	2
Górniak et al.(61)	YES	NO	NO	N/A	YES	YES	NO	N/A	3

Not applicable (N/A)
